# Application of the international league against rheumatism classification criteria for systemic juvenile idiopathic arthritis as a prognostic factor in patients with adults-onset Still’s disease

**DOI:** 10.1186/s12969-018-0225-1

**Published:** 2018-01-25

**Authors:** Ji Won Yang, Eunyoung Lee, Ji-Yeon Seo, Ju-Yang Jung, Chang-Hee Suh, Hyoun-Ah Kim

**Affiliations:** 1 Department of Rheumatology, Ajou University School of Medicine, 164 Worldcup-ro, Yeongtong-gu, Suwon, 16499 South Korea; 2 Department of Biomedical Informatics, Ajou University School of Medicine, 164 Worldcup-ro, Yeongtong-gu, Suwon, 16499 South Korea

**Keywords:** Adult-onset Still’s disease, Systemic juvenile idiopathic arthritis, Yamaguchi criteria, ILAR criteria

## Abstract

**Background:**

Adult-onset Still’s disease (AOSD) is an adult form of systemic juvenile idiopathic arthritis (JIA) that differs from the latter in its classification. This study evaluated the concordance between the International League Against Rheumatism (ILAR) criteria for systemic JIA and the Yamaguchi criteria and then compared their possible prognostic value in patients with AOSD.

**Methods:**

In a retrospective review of 169 adults with suspected AOSD, patients were classified according to the Yamaguchi or ILAR criteria. Then the concordance in cross-referencing the other group with the different criteria was investigated and the sensitivity and specificity of each set of criteria were determined. Disease activity markers in AOSD patients were correlated with positivity according to both systems.

**Results:**

Concordance was good in patients with suspected AOSD (k = 0.7144, *p* <  0.001) and low in those with a diagnosis of AOSD (k = 0.3787, *p* <  0.001). The sensitivity of the ILAR criteria in AOSD patients was 0.8864 (95% confidence interval (CI): 0.8322–0.9405), and the specificity was 0.7838 (0.6511, 0.9164). Positivity according to the ILAR criteria correlated with the systemic score (*r* = 0.763, *p* <  0.0001) and C-reactive protein levels (*r* = 0.183, *p* = 0.0356) and was associated with a relapse (odds ratio: 1.589, 95% CI: 1.043–2.421), macrophage activation syndrome (MAS; odds ratio: 1.993, 95% CI: 1.218–3.263) and care in the intensive care unit (ICU; odds ratio: 2.087, 95% CI: 1.086–4.011).

**Conclusions:**

In AOSD patients, there is fair concordance between the Yamaguchi and ILAR criteria for systemic JIA. Positive ILAR criteria may be useful for identifying AOSD patients at high risk for relapse, MAS and the need for ICU care. Further studies including larger populations from several centers are needed to confirm our results regarding the utility of the ILAR criteria in AOSD patients.

## Background

Still’s disease is a systemic form of juvenile idiopathic arthritis (JIA), the etiology and pathogenesis of which are unknown. Systemic JIA is a cause of fever of unknown origin (FUO) and it is accompanied by several systemic manifestations, such as arthritis, an evanescent rash, and serositis. A cohort of 14 adults who presented with the same symptoms as JIA patients was described in 1971, and the name adult-onset Still’s disease (AOSD) was proposed [1]. Although the adult and pediatric diseases are considered parts of the same spectrum, the diagnosis of one or the other depends on the age at onset. Individuals presenting at the age of 16 years and older are diagnosed with AOSD; younger individuals are diagnosed with systemic JIA [2, 3]. However, there are differences between JIA and AOSD, such as a higher seasonality in the former and a higher rate of pharyngitis in the latter [4]. A recent study compared the cytokine profiles of patients with AOSD and systemic JIA [5]. Among the shared features was a significant increase in interleukin 18 (IL-18) levels. Differences between the IL-6- and IL-18-based cytokine profiles may be responsible for the different clinical manifestations of JIA and AOSD and suggest the presence of two distinct subgroups within a single disease category [6].

AOSD is often difficult to diagnose because patients may present with several nonspecific symptoms and there are no serologic biomarkers of the disease. While there are several sets of clinical criteria for the classification of AOSD, those of Yamaguchi are used most widely and consist of fever, arthralgia, typical rash, and leukocytosis as major criteria, and sore throat, lymphadenopathy or splenomegaly, liver dysfunction, and the absence of rheumatoid factor (RF) and antinuclear antibody (ANA) as minor criteria [7]. Some manifestations are related to an unfavorable outcome after a chronic disease course and a prolonged period of time until clinical remission, such as typical rash, root joint arthritis, and polyarthritis [8].

AOSD and systemic JIA are considered similar diseases with a shared pathogenesis and gene expression profile, but they are difficult to classify according to the criteria currently available [5, 7, 9]. Among the various clinical criteria for diagnosing AOSD, the Yamaguchi criteria are commonly used in clinical practice, and are the most widely cited; however, they require the prior exclusion of neoplasms, infections, and autoimmune diseases mimicking AOSD [7, 10, 11]. The International League Against Rheumatism (ILAR) first proposed the term JIA, which includes all forms of arthritis that onset before the age of 16 years, persist for more than 6 weeks, and are of unknown origin [12, 13]. Although the ILAR classification of JIA is widely used, it has the limitation of being an incomplete system and new criteria have been proposed for the classification of JIA, including systemic onset [14, 15]. One study evaluated the efficacy of substituting adult diagnostic criteria in the evaluation of pediatric patients with suspected systemic JIA [16]. The results suggested that the Yamaguchi criteria can be useful for the subset of patients in the pre-arthritic phase of the disease. However, few studies have examined the utility of the ILAR criteria for the classification of patients with AOSD. Thus, in this retrospective study, we assessed the compatibility of the ILAR criteria for systemic JIA when applied to patients with AOSD. Conversely, we asked whether the ILAR criteria could be used to diagnose, evaluate disease activity, and predict the prognosis in patients with AOSD.

## Methods

### Patients

We identified 191 patients within the Ajou University Hospital computer system with the diagnostic code for AOSD; 22 of these patients were excluded from this study because of missing or insufficient data. We retrospectively reviewed 169 patients with suspected AOSD at the time of the initial visit to Ajou University Hospital between 2001 and 2017. During the diagnostic evaluation, 37 patients were diagnosed with another disease, including viral infection, palindromic rheumatism, lupus-like disease, and Kikuchi’s disease. Inclusion in the diagnosis of AOSD required (1) a diagnosis code of AOSD retained by the referring physician at the last follow-up; (2) a diagnosis of AOSD retained by the investigator (H.-A. K.); and (3) meeting none of the classification criteria for other immune-mediated inflammatory diseases previously described [10]. The 132 remaining patients with a diagnosis of AOSD were compared with the 37 patients with other diagnoses. All enrolled patients with five or more of the Yamaguchi criteria (and fulfilling at least two major criteria) were classified after excluding infections, malignancies (especially malignant lymphoma), and other rheumatic diseases [7]. The major and minor Yamaguchi criteria are as follows. The major criteria are (1) fever of at least 39 °C for at least 1 week, (2) arthralgia or arthritis for at least 2 weeks, (3) non-pruritic salmon-colored rash on the trunk/extremities, and (4) granulocytic leukocytosis (10,000/mL or greater). The minor criteria are (1) sore throat, (2) lymphadenopathy, (3) hepatomegaly or splenomegaly, (4) abnormal liver function tests, and (5) negative in tests for RF and ANA. Then, these patients were reclassified using the ILAR criteria for systemic JIA [12] and the concordance between the ILAR and Yamaguchi criteria was assessed. A daily fever exceeding 39 °C for at least 2 weeks or arthritis in one or more joints lasting for at least 6 weeks accompanied by one or more of the following: (1) evanescent erythematous rash, (2) generalized lymph node enlargement, (3) hepatomegaly or splenomegaly, and (4) serositis. We also evaluated the sensitivity and specificity of the ILAR criteria. This study was approved by the Institutional Review Board of our hospital (AJIRB-MED-MED-17-297).

### Variables

**Fig. 1:**
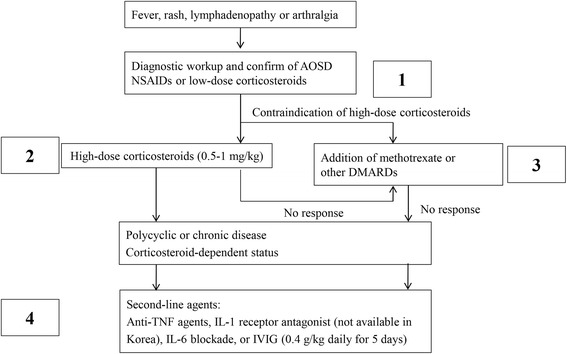
A diagram for 4-step treatment schedule in patients with adult-onset Still’s disease (AOSD). IVIG, intravenous immunoglobulin; IL-1, interleukin-1

All clinical data were retrieved from patient medical records stored in the hospital’s database. Clinical characteristics, including age, sex, clinical symptoms, follow-up period, organ involvement and extent, treatment, and outcome were evaluated. Each patient underwent a series of laboratory tests, including a complete blood count (CBC) and determination of the erythrocyte sedimentation rate (ESR) and levels of C-reactive protein (CRP), RF, ANA, ferritin, albumin, lactate dehydrogenase (LDH), and complement factors. AOSD disease activity was evaluated using the method of Pouchot et al., which assigns a score from 0 to 12 and adds 1 point for each of the following manifestations: fever, typical rash, pleuritis, pneumonia, pericarditis, hepatomegaly or abnormal liver function tests, splenomegaly, lymphadenopathy, leukocytosis ≥15,000/mm^2^, sore throat, myalgia, and abdominal pain [8]. Patients were treated in four steps: 1) patients used only low-dose corticosteroids or nonsteroidal anti-inflammatory drugs (NSAIDs); 2) high-dose corticosteroids without disease-modifying antirheumatic drugs (DMARDs); 3) DMARDs used regardless of the use of corticosteroids; and 4) patients were treated with corticosteroids, as well as tumor necrosis factor (TNF) inhibitors, IL-6 blockade or intravenous immunoglobulin (IVIG), regardless of the use of DMARDs (Fig. 1).

### Statistical analysis

The data are expressed as the mean ± standard deviation (SD). A *P* value <  0.05 was considered to indicate statistical significance. The sensitivity and specificity of the two sets of diagnostic criteria were tested in patients diagnosed with AOSD group and in the non-AOSD group. The agreement between the Yamaguchi and ILAR criteria was assessed according to the kappa (k) value: a k-value > 0.8 indicated excellent agreement, 0.61–0.8 meant good agreement, 0.21–0.4 indicated fair agreement, and < 0.2 was considered poor agreement [17]. A multivariate analysis of the significant markers was performed to determine those that were independently associated with the prognosis of AOSD. A Pearson correlation test was used to determine the correlation between ESR, CRP, ferritin, and leukocyte counts, as disease activity markers, and the Yamaguchi and ILAR criteria. All statistical analyses were two-sided and were carried out using SAS statistical software, ver. 9.4 (SAS Institute).

## Results

### Characteristics of the study population

Table 1 shows the clinical characteristics of the 169 patients seen at the hospital for suspected AOSD. Within this group, 132 patients were diagnosed with AOSD and 37 were diagnosed with another disease. In total, 12 patients were diagnosed with palindromic rheumatism, 11 with lupus-like disease, 4 with viral exanthema, 2 each with drug eruptions, Kikuchi’s disease, and systemic lupus erythematosus, and 1 each with breast cancer, thyroid cancer, human immunodeficiency virus infection, and hypereosinophilic syndrome. The main clinical features of the 169 patients were fever (98%), maculopapular skin rash (76%), arthralgia (89%), and arthritis (48%), which appeared more frequently in the AOSD group than in non-AOSD patients. The frequencies of other clinical symptoms, such as sore throat and lymphadenopathy, were not significantly different between the two groups. Mean leukocyte and neutrophil counts were significantly higher in AOSD patients (*p* <  0.001), who also had higher ferritin (*p* = 0.002), ESR (*p* <  0.001), and CRP (*p* < 0.001) levels. Patients with AOSD were treated with corticosteroids (95%), nonsteroidal anti-inflammatory drugs (83%), methotrexate (52%), hydroxychloroquine (31%), IVIG (19%), a TNF inhibitor (6%), and IL-6 blockade (3%). During the follow-up period, 35 patients (27%) had disease relapse, 11 patients (8%) were treated in the intensive care unit (ICU), and 4 (3%) died (Table 2). Of the 134 AOSD patients, 17 (13%) were diagnosed with macrophage activation syndrome (MAS). Three patients died of pneumonia with sepsis during the treatment, and one patient died of fulminant hepatitis related to AOSD.

**Table 1:** Demographic and baseline characteristics of patients with suspected adult-onset Still’s disease (AOSD)

Characteristics	AOSD suspected, but not diagnosed(*N* = 37)	AOSD patients(*N* = 132)	*P*-value
Age (year)	39.4 ± 12.8	41.9 ± 15.9	0.37
Sex (Male)	15 (41%)	38 (29%)	0.17
Fever	29 (78%)	130 (98%)	<.001
Sore throat	11 (30%)	71 (54%)	0.010
Maculopopular skin rash	12 (32%)	102 (77%)	<.001
Lymphadenopathy	5 (14%)	45 (34%)	0.015
Splenomegaly	4 (11%)	29 (22%)	0.130
Hepatomegaly	2 (5%)	17 (13%)	0.200
Pericarditis	0 (0%)	11 (8%)	0.069
Pleuritis	0 (0%)	11 (8%)	0.069
Arthralgia	28 (76%)	117 (89%)	0.046
Arthritis	9 (24%)	64 (48%)	0.009
Hemoglobin, g/dL	12.5 ± 0.2	11.4 ± 0.1	<.001
Leukocytes, /μL	7295.1 ± 523.5	14,708.8 ± 599.3	<.001
Neutrophils, /μL	4714.7 ± 451.3	12,734.8 ± 594.8	<.001
lymphocyte, /μL	1838.0 ± 154.6	1100.9 ± 48.0	<.001
Platelets, × 10^3^ μL	274.0 ± 16.2	301.1 ± 11.8	0.26
ESR, mm/h	40.4 ± 5.2	64.8 ± 2.6	<.001
CRP, mg/dL	4.3 ± 1.1	11.6 ± 0.7	<.001
AST, mg/dL	56.5 ± 17.4	93.3 ± 17.4	0.065
ALT, mg/dL	66.7 ± 21.7	94.2 ± 21.7	0.28
Ferritin, mg/dL	721.4 ± 246.0	6007.6 ± 887.3	0.002
LDH, mg/dL	334.0 ± 52.8	593.5 ± 103.0	0.21
ANA positivity	8 (29%)	21 (17%)	0.16
RF positivity	3 (9%)	10 (8%)	0.85
Systemic score	2.6 ± 0.2	5.0 ± 0.1	<.0001

*AOSD* adult-onset Still’s disease, *ESR* erythrocyte sedimentation rate, *CRP* C-reactive protein, *AST* aspartate transaminase, *ALT* alanine transaminase, *LDH* lactate dehydrogenase, *ANA* antinuclear antibody, *RF* rheumatoid factor. All values are presented as the number (%) or the mean ± SE except the age as the mean ± SD. Systemic scores (0–12) were obtained using the method described by Pouchot et al. [7], with 1 point assigned for each of the following manifestations: fever, typical rash, pleuritis, pneumonia, pericarditis, hepatomegaly or abnormal liver function tests, splenomegaly, lymphadenopathy, leukocytosis ≥ 15,000/mm^2^, sore throat, myalgia, and abdominal pain

**Table 2:** Treatment and prognosis of the patients with adult-onset Still’s disease (AOSD) during follow-up

	AOSD patients(*N* = 132)		AOSD patients(*N* = 132)
Medication		Prognosis	
NSAIDs	109 (83%)	Relapse	35 (27%)
Corticosteroid	126 (95%)	MAS	17 (13%)
DMARD	88 (67%)	ICU	11 (8%)
HCQ	41 (31%)	Death	4 (3%)
Azathioprine	18 (14%)	Treatment step	
Sulfasalazine	11 (8%)	1	1 (1%)
Leflunomide	7 (5%)	2	27 (20%)
Methotrexate	69 (52%)	3	70 (53%)
IVIG	25 (19%)	4	34 (26%)
TNF inhibitor	8 (6%)		
IL-6 blockade	4 (3%)		

*AOSD* adult-onset Still’s disease, *NSAIDs* nonsteroidal anti-inflammatory drugs, *DMARD* disease modifying antirheumatic drugs, *HCQ* hydroxychloroquine, *IVIG* intravenous immunoglobulin, *TNF* tumor necrosis factor, *IL-6* interleukin-6, *MAS* macrophage activation syndrome, *ICU* intensive unit care. Values are expressed as *n* (%)

### Concordance between Yamaguchi and ILAR criteria in patients with suspected AOSD

Concordance between the Yamaguchi and ILAR criteria in patients with suspected AOSD was good (k = 0.7144, *p* < 0.001) whereas in the diagnosed AOSD group it was fair (k = 0.3787, *p* < 0.001), and lower than that based on a comparison with all suspected AOSD patients (Table 3). Thus, overall, there was substantial concordance between the two sets of criteria. We evaluated the sensitivity and specificity of the ILAR criteria for systemic JIA in patients with suspected AOSD. The sensitivity was 0.8864, with a 95% confidence interval (CI) of 0.8322–0.9405, and the specificity was 0.7838, with a 95% CI of 0.6511–0.9164 (Table 4).

**Table 3:** Agreement between the Yamaguchi and International League Against Rheumatism (ILAR) criteria in patients with suspected adult-onset Still’s disease (AOSD) or with confirmed AOSD

	Yamaguchi and ILAR criteria
All suspected AOSD	
Kappa (*p*-value)	0.7144 (< 0.0001)
95% confidence interval	(0.5947, 0.8341)
AOSD-diagnosed patients	
Kappa (*p*-value)	0.3787 (< 0.0001)
95% confidence interval	(0.1416, 0.6159)

The concordance between the Yamaguchi and ILAR criteria was assessed by the kappa (k) value: a k-value > 0.8 indicated excellent agreement, 0.61–0.8 meant good agreement, 0.21-0.4 indicated fair agreement, and < 0.2 was considered poor agreement

**Table 4:** Sensitivity and specificity of International League Against Rheumatism (ILAR) and Yamaguchi criteria in suspected adult-onset Still’s disease (AOSD) patients

Disease	ILAR criteria		Yamaguchi criteria		
	+	–	+	–	Total
AOSD patients	117	15	119	13	132
Not AOSD patients	8	29	3	34	37
Total	125	44	122	47	169

The sensitivity was 88.6%, and the specificity was 78.4%, with the ILAR criteria. The sensitivity was 90.2%, and the specificity was 91.9%, with the Yamaguchi criteria

### Correlation between disease activity markers and JIA and Yamaguchi criteria positivity in AOSD patients

We evaluated correlations between the levels of disease activity markers, such as the systemic score, ESR, and CRP, and the number of positive JIA or Yamaguchi criteria in patients with AOSD. The systemic score (*ρ* = 0.437, *p* < 0.0001), leukocyte count (*ρ* = 0.312, *p* = 0.0003), ESR (*ρ* = 0.178, *p* = 0.0406), CRP level (*ρ* = 0.216, *p* = 0.0128), and LDH level (*ρ* = − 0.307, *p* = 0.0056) correlated positively and significantly with the Yamaguchi criteria. The relationships between the ILAR criteria for systemic JIA and the systemic score (*ρ* = 0.741, *p* < 0.0001), CRP level (*ρ* = 0.237, *p* = 0.0063), and albumin (*ρ* = − 0.281, *p* = 0.0011) were also significant and positive (Table 5).

**Table 5:** Correlation between disease activity markers and the number of positive International League Against Rheumatism (ILAR) and Yamaguchi criteria in AOSD patients

	Yamaguchi criteria		ILAR criteria	
	*ρ*	*P*-value	*ρ*	*P*-value
Systemic score	0.437	< 0.0001	0.741	< 0.0001
Leukocytes	0.312	0.0003	−0.007	0.9369
Hemoglobin	−0.062	0.4802	−0.092	0.2953
Platelets	0.155	0.0764	−0.102	0.2461
ESR	0.178	0.0406	0.115	0.1890
CRP	0.216	0.0128	0.237	0.0063
Ferritin	0.169	0.0534	0.055	0.5274
LDH	−0.307	0.0056	−0.055	0.6290
Total bilirubin	−0.150	0.0864	−0.002	0.9798
Albumin	−0.156	0.0744	−0.281	0.0011
AST	−0.104	0.2334	−0.035	0.6891
ALT	−0.032	0.7151	−0.058	0.5064

*ESR* erythrocyte sedimentation rate, *CRP* C-reactive protein, *LDH* lactate dehydrogenase, *AST* aspartate aminotransferase, *ALT* alanine aminotransferase. Systemic scores (0–12) were obtained using the method described by Pouchot et al. [8], with 1 point assigned for each of the following manifestations: fever, typical rash, pleuritis, pneumonia, pericarditis, hepatomegaly or abnormal liver function tests, splenomegaly, lymphadenopathy, leukocytosis ≥ 15,000/mm^2^, sore throat, myalgia, and abdominal pain. The Pearson correlation statistic was used to compare the correlation between ESR, CRP, ferritin, and leukocyte counts, as disease activity markers, and the Yamaguchi and ILAR criteria

### Positivity of the ILAR criteria for systemic JIA and the Yamaguchi criteria as a prognostic factor in AOSD patients

The frequencies of relapse, MAS, care in the ICU, and death were designated as prognostic factors and then the data were analyzed using the logistic regression model with the positivity of the ILAR and Yamaguchi criteria in patients with AOSD. None of the prognostic factors were significantly associated with the Yamaguchi criteria but the relationships of relapse (odds ratio = 1.712, 95% CI = 1.166–2.512), MAS (odds ratio = 1.993, 95% CI = 1.218–3.263) and ICU care (odds ratio = 2.080, 95% CI = 1.135–3.811) with the positivity of the ILAR criteria for systemic JIA in patients with AOSD were significant (Table 6).

**Table 6:** Positivity of the International League Against Rheumatism (ILAR) criteria and Yamaguchi criteria as a prognostic factor for death or care in the intensive care unit (ICU) in patients with adult-onset Still’s disease (AOSD). The data were analyzed using a logistic regression model

Outcome of interests	Effect					
	Positive Yamaguchi criteria			Positive ILAR criteria		
	Odds ratio estimate	95% Wald confidence interval		Odds ratio estimate	95% Wald confidence limits	
Prognosis						
Relapse	1.295	0.729	2.298	1.589	1.043	2.421
MAS	1.325	0.750	2.340	1.993	1.218	3.263
ICU	1.145	0.464	2.827	2.087	1.086	4.011
Death	0.769	0.217	2.726	0.746	0.236	2.364

*AOSD* adult-onset Still’s disease, *MAS* macrophage activation syndrome, *ICU* intensive unit care

## Discussion

This is the first study to evaluate the ILAR criteria for systemic JIA in patients with AOSD. The results showed fair concordance between the Yamaguchi and ILAR criteria for systemic JIA in patients with AOSD. However, the positivity of the ILAR criteria may be useful for the identification of AOSD patients at high risk for relapse, association with MAS and ICU care.

Several studies have compared children and adults with Still’s disease [18–20], but most have been unable to find specific differences between AOSD and systemic JIA. One study showed that nine patients with systemic JIA met the Yamaguchi criteria [19], while another detected differences with respect to articular involvement between the two groups [18]. Nonetheless, given the large number of similarities between pediatric and adult patients, the diseases can be considered to the same or at least similar. Therefore, we evaluated the sensitivity and specificity of the ILAR criteria for systemic JIA in patients with suspected AOSD. Our results showed concordance between the Yamaguchi and ILAR criteria in these patients. Among the 169 patients with suspected AOSD, Cohen’s kappa was 0.7144, indicative of substantial agreement between the two sets of criteria. When only the 132 AOSD patients were considered, Cohen’s kappa was lower (0.387), but it still supported agreement between the Yamaguchi and ILAR criteria. There was substantial concordance when the entire patient population with suspected AOSD was included, but only fair concordance when this was limited to the AOSD group. This is probably due to the fact that arthritis was more common in the AOSD group than in the non-AOSD group, but it was still present in less than 50% of cases. This may reflect the ILAR criteria requiring the presence of arthritis to diagnose a patient with systemic JIA [12]. One study has suggested that the presence of arthritis is a strict criterion, but this could lead to an unacceptable delay in diagnosis [16]. That study also showed that patients with systemic JIA may experience a significant period during which they do not have arthritis, particularly at disease onset [16]. Those authors concluded that the Yamaguchi criteria may be useful for the subset of patients in the pre-arthritic phase of the disease. As part of this process, Dr. Martini proposed a prospective research plan for establishing a new provisional classification system for JIA in the 23rd European Pediatric Rheumatology Congress [15]. In this study, our patients with AOSD had a low frequency of arthritis, which may explain the low sensitivity and specificity of the ILAR criteria in patients with AOSD.

Many recent studies have sought to identify simple markers for evaluating AOSD disease severity, activity, or prognosis. The candidate biomarkers included simple clinical laboratory markers, such as ESR, CRP, ferritin, the neutrophil to lymphocyte ratio, and procalcitonin, as well as several cytokines or chemokines, such as IL-6, IL-8, CXC motif chemokine 13, and IL-18 [5, 21–23]. However, clinical laboratory markers are relatively nonspecific, and cytokine determinations are not clinically feasible. A recent study suggested that Pouchot’s systemic score could predict a poor outcome in patients with AOSD. Thus, a score > 7 and the presence of any complication, such as MAS, kidney failure, or myocarditis at diagnosis, were associated with mortality [24]. Our study evaluated the positivity of the ILAR criteria in patients with AOSD, with respect to preexisting disease activity markers and prognosis. ILAR criteria positivity correlated with the systemic scores and the levels of CRP and albumin. We were also able to demonstrate an association between positivity for the ILAR criteria and two prognostic factors, relapse and ICU care, in patients with AOSD. These results suggest that the ILAR criteria can aid the evaluation of disease activity and prediction of prognosis of AOSD. The differences between the ILAR and Yamaguchi criteria are the presence of serositis and absence of leukocytosis, sore throat, and elevated liver enzyme concentrations. Our results could be related to the presence of serositis in the ILAR criteria. Some studies suggested that serositis is a poor prognostic factor for early onset JIA or joint damage in systemic JIA [25, 26].

There were several important limitations to this study. First, because it was conducted in a single center, the data were limited and may have been subject to selection bias. Second, because we reviewed electronic medical records, some patient data may have been missing and the records of a few patient records were not available. Third, the patients who did not meet the initial screening criterion regarding the diagnostic code for AOSD were not included.

## Conclusion

Despite the lack of sensitivity and specificity of the ILAR criteria for systemic JIA in the classification of AOSD, there was fair concordance between the Yamaguchi and ILAR criteria in AOSD patients. Our results indicate that the ILAR criteria can be used as an additional predictor of relapse, MAS and ICU care in AOSD patients. Further studies of larger populations from several centers are needed to confirm our results regarding the utility of the ILAR criteria in AOSD patients.

## Abbreviations

ALT: Alanine transaminase
ANA: Antinuclear antibody
AOSD: Adult-onset Still’s disease
AST: Aspartate transaminase
CBC: Complete blood count
CI: Confidence interval
CRP: C-reactive protein
DMARD: Disease-modifying antirheumatic drugs
ESR: Erythrocyte sedimentation rate
FUO: Fever of unknown origin
HCQ: Hydroxychloroquine
ICU: Intensive unit care
IL-18: Interleukin 18
ILAR: International League Against Rheumatism
IVIG: Intravenous immunoglobulin
JIA: Juvenile idiopathic arthritis
k: kappa
LDH: Lactate dehydrogenase
RF: Rheumatoid factor
SD: Standard deviation
SE: Standard error
TNF: Tumor necrosis factor
